# A systematic review on the contribution of DNA methylation to hearing loss

**DOI:** 10.1186/s13148-024-01697-9

**Published:** 2024-07-05

**Authors:** Vibha Patil, Patricia Perez-Carpena, Jose A. Lopez-Escamez

**Affiliations:** 1 Meniere’s Disease Neuroscience Research Program, Faculty of Medicine and Health, School of Medical Sciences, The Kolling Institute, University of Sydney, Rm 611024, Level 11 Kolling Institute | 10 Westbourne St, St Leonards, Sydney, NSW 2064 Australia; 2 Division of Otolaryngology, Department of Surgery, Instituto de Investigación Biosanitaria, Ibs.Granada, Universidad de Granada, Granada, Spain; 3 Otology & Neurotology Group CTS495, Instituto de Investigación Biosanitaria, ibs.GRANADA, Universidad de Granada, Granada, Spain; 4 Sensorineural Pathology Program, Centro de Investigación Biomédica en Red en Enfermedades Raras, CIBERER, Madrid, Spain; 5 Department of Otolaryngology, Hospital Universitario San Cecilio, Instituto de Investigacion Biosanitaria, ibs.GRANADA, Granada, Spain

**Keywords:** Sensorineural hearing loss, Age-related hearing loss, Gene regulation, CpG methylation

## Abstract

**Background:**

DNA methylation may have a regulatory role in monogenic sensorineural hearing loss and complex, polygenic phenotypic forms of hearing loss, including age-related hearing impairment or Meniere disease. The purpose of this systematic review is to critically assess the evidence supporting a functional role of DNA methylation in phenotypes associated with hearing loss.

**Results:**

The search strategy yielded a total of 661 articles. After quality assessment, 25 records were selected (12 human DNA methylation studies, 5 experimental animal studies and 8 studies reporting mutations in the *DNMT1* gene). Although some methylation studies reported significant differences in CpG methylation in diverse gene promoters associated with complex hearing loss phenotypes (ARHI, otosclerosis, MD), only one study included a replication cohort that supported a regulatory role for CpG methylation in the genes *TCF25* and *POLE* in ARHI. Conversely, several studies have independently confirmed pathogenic mutations within exon 21 of the *DNMT1* gene, which encodes the DNA (cytosine-5)-methyltransferase 1 enzyme. This methylation enzyme is strongly associated with a rare disease defined by autosomal dominant cerebellar ataxia, deafness and narcolepsy (ADCA-DN). Of note, rare variants in *DNMT1* and *DNMT3A* genes have also been reported in noise-induced hearing loss.

**Conclusions:**

Evidence supporting a functional role for DNA methylation in hearing loss is limited to few genes in complex disorders such as ARHI. Mutations in the *DNMT1* gene are associated with ADCA-DN, suggesting the CpG methylation in hearing loss genes deserves further attention in hearing research.

**Supplementary Information:**

The online version contains supplementary material available at 10.1186/s13148-024-01697-9.

## Introduction

Hearing loss in humans is one of the major burdens of disease worldwide [1]. Sensorineural hearing loss (SNHL) is the most common type, and it results from abnormal sound processing in the organ of Corti, the auditory pathway or auditory cortex. According to its etiology, SNHL is classified as genetic SNHL and acquired SNHL. Most non-syndromic genetic deafness are monogenic disorders and their inheritance can be autosomal dominant, recessive, X-linked or mitochondrial [2]. Conversely, age-related hearing loss (presbycusis) or noise-induced hearing loss (NIHL) is defined by a progressive course involving initially high frequencies and is considered multifactorial conditions with an environmental origin (i.e., vascular risk factors or noise exposure) [3].

Familial segregation and sequencing studies have been invaluable in developing our understanding of monogenic SNHL since a genetic component is present in ~ 50% of all hearing loss cases. However, the underlying molecular mechanisms of acquired SNHL remain poorly elucidated [4]. While genome-wide association studies (GWAS) in adults affected by hearing loss continue to discover new candidate genes for hearing loss, a number of limitations to this approach have been identified. For example, while a genetic susceptibility may be highly relevant in specific types of hearing loss, it may not be the predominant factor for other types of hearing loss. It is challenging to make conclusions regarding etiological heterogeneities that encompass these large cohorts of self-reported hearing loss patients. Secondly, elucidating genomic mechanisms from the association in GWAS have proven difficult in past studies [4]. Developing a strategy that allows for elucidation of molecular mechanisms of hearing loss in its various etiologies is crucial to the development of effective treatment strategies.

Emerging evidence is suggesting that DNA methylation may also have an important regulatory role in hearing loss and its associated conditions [5]. DNA methylation is an epigenetic modification where a cytosine residue is converted to 5-methylcytosine (5mC) by DNA methyltransferases (DNMTs). Although the majority of methylation in human somatic cells is observed within a CpG dinucleotide context (within ~ 70% of gene promoters), it has also been identified within CpA, CpC and CpT contexts collectively known as non-CpG methylation [6]. Both CpG and non-CpG methylation can silence gene expression by preventing transcription factor binding or through the recruitment of repressive complexes [6]. Hence DNA methylation can lead to phenotypic changes without altering the underlying DNA sequence.

This systematic review aims to consolidate current literature linking DNA methylation and hearing loss in order to highlight remaining gaps in knowledge which may help elucidate a fuller comprehension of epigenetic changes in common and rare disorders associated with hearing loss. Understanding the precise mechanisms and specific genes involved in hearing function, which may be regulated through DNA methylation, could lead to the development of more refined studies that can help produce new therapeutic strategies for preventing or treating hearing loss and its associated conditions.

## Materials and methods

### Study design

This review followed the PRISMA guidelines (Preferred Reported Items for Systematic Reviews and Meta-Analyses) [7] and adhered to the MOOSE checklist (Meta-analyses Of Observational Studies in Epidemiology) [8]. The review protocol was also registered on PROSPERO (CRD42023440491).

According to the methodology established for systematic reviews, the PICO question included the following items:

- Participants: Patients or animal models with hearing loss
- Intervention or variables of interest (Exposure): epigenetic or epigenomic studies profiling DNA methylation
- Controls: controlled and uncontrolled studies
- Main results: regions or genes with differentially methylated cytosines (CpG)
- Secondary outcomes: predicted pathways associated with hearing loss.
- Study design: Case–control studies, twin studies, animal models with hearing loss.

### Search strategy

The search, conducted on November 15, 2023, used PubMed, Scopus and Cochrane databases with the following MesH terms: *(hearing loss OR age-related hearing loss) AND (Cytosine OR methylation OR Epigenetics OR Epigenomics)*, and it was limited to original articles, published from the year 2000 onward. Replicates in references were removed, and articles incongruent with the review's objectives were omitted through the screening of their titles and abstracts. This process resulted in the retention of solely those records that conformed to the predefined inclusion criteria. In addition, the following exclusion criteria were used:

- Studies that did not include any audiological assessments.
- Studies published in other languages than English.
- Single-case reports, except multicase family or twin studies.

### Data collection

Two different reviewers (V.P, P.P–C) independently extracted study characteristics and outcomes from all the included studies, and data were compared. A third reviewer (J.A.L.E) was consulted when a consensus could not be reached. Data pertaining to the review's objective were extracted from each article. From each study, the data collected included reference information (author and year of publication), geographical location, study design, research objectives, sample size, gender distribution, average age and the primary findings for each study (differentially methylated regions, DMR or genes, DMG).

### Data synthesis/summary

We compiled the DMR and DMG across different studies for each condition or disease associated with hearing loss. We also summarized the studies involving mutations in the *DNMT1* gene.

### Analysis of subsets/subgroup

Studies were further subgrouped into three categories, (i) human studies of hearing loss and methylation, (ii) animal studies of hearing loss and methylation, (iii) hearing loss and DNMT1 mutations. All studies encompassed standardized audiometric testing for hearing loss in humans; auditory brainstem response in animals OR had a confirmed diagnosis of a disease where hearing loss is essential to pathophysiology.

### Quality and risk of *bias* assessment

Was also evaluated; the ROBINS-E tool was used in non-randomized Studies of Exposures [9]. These tools consist of seven domains, namely: (1) confounding-induced bias, (2) bias in exposure measurement, (3) bias in participant selection for the study, (4) bias resulting from post-exposure interventions, (5) bias due to missing data, (6) bias in outcome measurement and (7) bias in the selection of reported results. Notably, domain 4 was deemed irrelevant for this review and was consequently excluded. The assessed risk of bias varied from "Low" to "Moderate," "High" or "Very High." Overall bias risk was determined by evaluating all domains collectively. A color-coded scale (white for not applicable, green for Low risk, yellow for Moderate risk, red for High risk and black for Very High risk) was employed to present a concise summary, as detailed in Table S1.

The SYRCLE’s risk of bias tool was used to assess animal studies [10] and included in Table S2. This tool contains 10 entries, which are related to 6 types of bias (selection bias, performance bias, detection bias, attrition bias, reporting bias and other biases), and helps to define the level of risk of bias based on several specific question for each domain. According to this, the risk of bias has been stablished as low/high/unclear.

## Results

**Fig. 1:**
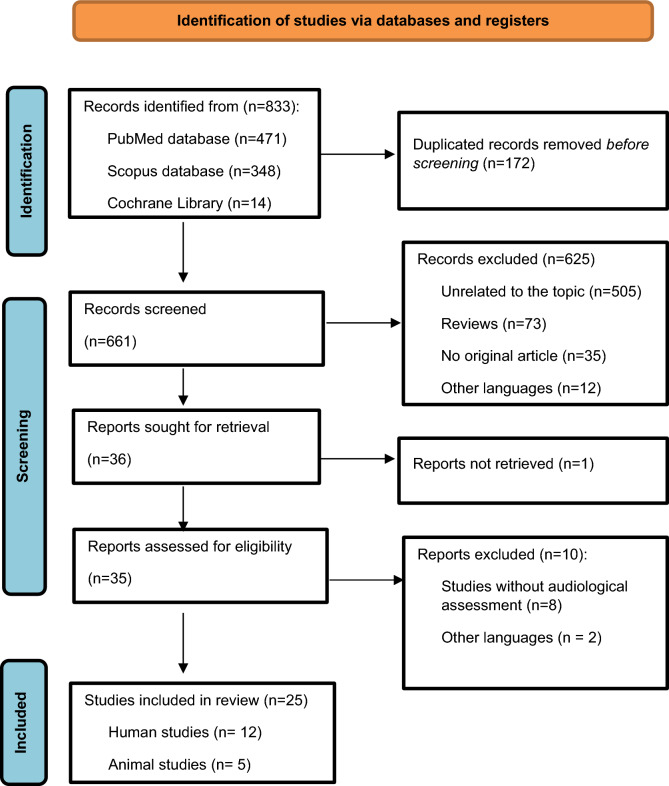
Flow diagram for the DNA methylation study selection

We selected a total of 25 articles which fit the inclusion criteria, 12 human DNA methylation studies, 5 experimental animal studies in mice [3] and rats [2] and 8 studies reporting mutations in the *DNMT1* gene. Figure 1 details the flowchart for selection of the included articles.

Of the 12 human hearing loss studies, one study was conducted in relation to environmental exposure to Pb and Cd in children, whereas 11/12 were conducted in patients with concurrent presence of a relevant pathology namely, age-related hearing loss (ARHL, 6 studies), otosclerosis (OTSC), ototoxicity, Meniere's disease (MD) and diabetic-related hearing loss (DRHL) in a case–control setting (Tables 1, 2). Only one out of all human hearing loss studies (1/12) included a replication cohort in their study design.

**Table 1:** Descriptive features of selected studies which investigated DNA methylation in relation to hearing loss. All studies contain an element of confirmed diagnosis of hearing loss or audiometric tests

Human studies									
Author/Year	Country	Disease	Design	Covariates	Sample Size	Sex/Age	Main Objective	Results	Rep
Bouzid, A., et al. 2022 [1]	Tunisia	OTSC	Case–control. Genotyping: Cross-sectional study of SNP & Q-MSP		*n* = 229; case = 109; control = 120	No info	Explore association of rs1021188 SNP in *TNFSF11* gene with OTSC subjects and to assess if promoter DNA methylation of *TNFSF11* reveals susceptibility to OTSC	*TNFSF11* polymorphism rs1021188 is associated with OTSC. Statistically significant differential methylation detected in *TNFSF11* CpG Island promoter region between OTSC case vs controls. Overall, less than 5% variation found. 4.4-fold decrease in methylation across region in case vs control	No
Bouzid, A., et al. 2018 [2]	Tunisia	ARHL	Case–control. Q-MSP		*n* = 50; case = 25; control = 25	F Only /50–75	Investigate if DNA methylation within *CDH23* intragenic CpG Island could be a risk factor associated to age-related hearing impairment (ARHI)	*CDH23* intragenic CpG Island has statistically significant 3.27-fold higher methylation in ARHI compared to normal. Less than 5% variation detected	No
Bouzid, A., et al. 2018 [3]	Tunisia	ARHL	Case–control. RRBS & Gene Expression Microarray		*n* = 18; case = 9; control = 9	F Only /No info	Investigate if DNA methylation can identify novel biomarkers for ARHI	*P2RX2*, *KCNQ5, ERBB3* and *SOCS3* genes are hypermethylated and downregulated in female subjects affected with presbycusis	No
Brown, A. L., et al. 2017 [4]	USA, Texas	Ototoxicity	450 K Methylation Array		*n* = 62; rep = 18	no info	Identify genome-wide methylation sites that are associated with ototoxicity susceptibility	Methylation at site cg14010619 may modify *PAK4* gene expression	Yes
Flook, M., et al. 2021 [5]	Spain	MD	WGBS	No	*n* = 20; case = 14; control = 6	no info	Identify an MD methylation signature and potential disease mechanisms	Methylation patterns can distinguish MD patients from controls as well as MD patients with various levels of cytokines	No
Guo, L., et al. 2023 [6]	China	ARHL	RRBS		*n* = 122; case = 61; control = 61	No info /50 +	Explore differences in DNA methylation associated with ARHL	Detected 18, 42 and 12 CpG sites and 6, 11 and 6 DMRs associated with LFPTA, Speech-PTA and HFPTA, respectively. Identified pathways that may be potentially associated with hearing loss mechanism	No
Hao, J., et al. 2018 [7]	China	DRHL	RRBS		*n* = 10; case = 5; control = 5	No info /30–65	Investigate genome-wide DNA methylation of T2DM patients with and without hearing loss to identify loci susceptible to methylation changes in DRHL	Only *KCNJll* (a T2DM gene) was identified to align with one of the 38 DRMS detected. No hearing loss genes aligned to the DMRs. Methylation profiles may identify susceptible loci in DRHL	No
Kuo, P. L., et al. 2021 [8]	USA, Baltimore	ARHL	450 K Methylation Array	Yes	*n* = 236	F: 124; M: 112	Investigate relationship between epigenetic age acceleration and audiometric hearing	Some epigenetic age acceleration measurements, as determined by DNA methylation profiles, are associated with hearing	No
Lassaletta, L., et al. 2006 [9]	Spain	Vestibular schwannoma	Q-MSP		*n* = 22	F: 15; M: 7	Analyze relationship of DNA methylation profiles in a specified gene set with clinical and radiological features of Vestibular Schwannoma (VS)	Some significant correlations between the CpG methylation status and clinical and radiological features in VS. However larger prospective cohort required for statistical significance	No
Wolber, L. E., et al. 2014 [10]	UK	ARHL	27 K Methylation Array, 450 K Methylation Array		*n* = 115; replication = 203	F Only /41–86	Investigate the association between whole-genome DNA methylation from blood and hearing ability	Strong associations with DNA methylation in the promoters of 10 genes were identified, of which two (*TCF25* and *POLE*) were replicated in an independent cohort	Yes
Xu, J., et al. 2017 [11]	China	ARHL	Case–control. Pyrosequencing	No	*n* = 206; case = 102; control = 104	F: 46; M: 56	Investigate whether promoter methylation of *SLC26A4* associated with higher risk of hearing loss	Methylation of CpG sites within the *SLC26A4* gene reflects an increased risk of hearing loss in males	No
Xu, L., et al. 2020 [12]	China	Children exposed to Cd and Pb	Case series. WGBS	NA	*n* = 116	No info /3—7	Investigate if early life exposure to lead and cadmium levels and methylation at specific gene regions are associated with hearing loss	Lead exposure was significantly associated with hearing loss and statistically higher methylation at cg 02978827 in the promoter of *Rb1* as well CpG Sites located 14 bp upstream and 4 bp downstream of the promoter were identified	No

*OTSC* Osteosclerosis, *ARHL* Age-Related Hearing Loss, *MD* Meniere’s Disease, *DRHL* Diabetes-Related Hearing Loss, *T2DM* Type II Diabetes Mellitus, *Q-MSP* Quantitative Methylation-Specific PCR, *RRBS* Reduced Representation Bisulfite Sequencing, *WGBS* Whole-Genome Bisulfite Sequencing

**Table 2:** Descriptive features of human studies highlighting the gene symbols and gene regions where differential DNA methylation was observed as well as definitions of hearing loss used for each study

Human studies				
Author/year	All genes	Hearing loss genes	DNA methylation regions	Hearing loss phenotype
Bouzid, A., et al. 2022	*TNFSF11*		PromoterChr13:4257390442574865	Unspecified
Bouzid, A., et al. 2018	*CDH23*	*CDH23*	CpG island site of the junction exon 54intron 54 in CDH2	Hearing loss thresholds ≥ 20 dB
Bouzid, A., et al. 2018	*227 GENES IN TOTAL REPORTED*	*ASTN2*	Exon1 Chr9:119449474-119449519	Hearing loss defined by pure tone audiometry at 0.25 0.5 1 2 4 and 8 kHz frequencies
	*ATP2B3*		Promoter ChrX:152801006-152801151	
	*BTBD2*		Exon1 Chr19:2015326-2015433	
	*C19orf55*		Promoter Chr19:36247138-36247267	
	*CCDC85C*		Exon1 Chr14:10006965-100070237	
	*CEACAM1*		Promoter Chr19:43034432-43034565	
	*ERBB3*		Exon2 Chr19:677925-677973	
	*FSTL3*		Exon1 Chr5:75699163-75699285	
	*IQGAP2*		Exon1 Chr6:73332056-73332153	
	*KCNQ5*		Exon1 Chr17:4458422-4458556	
	*MYBBP1A*		Exon1 Chr17:7311711-3711829	
	*NLGN2*		Promoter Chr12:133195302-13319539	
	*P2RX2*		Exon1 Chr12:133195405-133195423	
	*PGP*		Exon2 Chr16:2261684-2262961	
	*RAB2B*		Promoter Chr14:21945595-2196532	
	*RUSC1*		Exon1 Chr1:155293868-155294305	
	*SOCS3*		Exon1_2Chr17:76355149-76355243	
	*TMED7-TICAM2*		Exon1 Chr5:114961500-114961615	
Brown, A. L., et al. 2017	*PAK4*		Chr19: cg14010619	Hearing loss defined by pure tone audiometry Grade 0 = < 20 dB Grade 1 = > 20 dB at ≥ 6 kHz Grade2 = > 20 dB at ≥ 4 kHz Grade 3 = > 20 dB at ≥ 2 kHz Grade 4 = > 40 dB at ≥ 2 kHz
Flook, M., et al. 2021	*H3Y1* *ACSBG1* *IL32*			Meniere Disease
Guo, L., et al. 2023	*C3*		chr19:6710806-6711077	Speech-Pure Tone Audiometry of ≥ 25 dB in the better ear was used to define hearing loss
	*TEX19*		chr17:80303573-80303880	
	*GBX2*		chr2:237071725-237072438	
	*CD247*		chr1:167408553-167408867	
	*SPATA18*		chr4:52942852-52943232	
	*ZCCHC8*		chr12:122983976-122984305	
	*CD247*		chr1:167408553-167408867	
	*TEX19*		chr17:80303573-80303880	
	*C3*		chr19:6710806-6711077	
	*MRGPRG-AS1*		chr11:3243153-3243455	
	*MKX*		chr10:28034352-28034507	
	*TMEM1 02*		chr17:7339626-7340311	
	*SPATA18*		chr4:52942852-52943232	
	*GRIN3B*		chr19:1008897-1009874	
	*LINC02249*		chr15:30517467-30517618	
	*PRDM16*		chr1:2990062-2990407	
	*OSR2*		chr8:99986099-99986645	
	*S100A13*		chr1:153606037-153606314	
	*RBMS2*		chr12:56882420-56882571	
	*ALG10*		chr12:34499106-34501260	
	*GPC5*		chr13:92051618-92051955	
	*NRN1*		chr6:6002421-6002736	
	*C21orf58*		chr21:47737945-47738279	
	*CD247*			
Hao, J., et al. 2018	*KCNJ11*		Not specified	Frequency range of 0.7 k–6 kHz for DPOAE Grade 0 = DPOAE meets SNR criteria normal range Grade 1 = DPOAE meets SNR criteria abnormal range Grade 2 = DPOAE does not meet SNR criteria
Kuo, P. L., et al. 2021	*N/A*	*N/A*	N/A	Pure tone audiometry 0.5– kHz Higher Pure Tone Audiometry = worse hearing
Lassaletta, L., et al. 2006	*RASSF1A* *RARB* *VHL* *PTEN* *HMLH1* *RB1* *ER* *TP16* *CASP8* *TIMP3* *MGMT* *DAPK* *TP73* *GSTP1* *TP14* *THBS*		Not specified	Pure Tone Average threshold of 25db – 62 dB recorded in all patients who displayed hearing loss symptoms
Wolber, L. E., et al. 2014	*TCF25* *PGM3* *CDO1* *NOC2L* *MYBPC3* *FGFR1* *POLE* *VPS2B* *HNRNPA* *APOCC4*		Not specified	Hearing loss defined by pure tone average for frequencies 0.125–8 kHz according to the recommendations of the British Society of Audiology
Xu, J., et al. 2017	*SLC26A4*	*SLC26A4*	Chr7:107300940107301001	Pure Tone Average greater than 60 dB hearing loss, control less than 26 dB
Xu, L., et al. 2020	*Rb1* *CASP8* *MeCP2*		chr13:48877561- 48877684 chr2:202097129- 202122658 chrX:153363708- 154097766	Pure Tone Average threshold above 25 dB considered as hearing loss

The methodological approach for these studies fit into two main categories, site-specific methylation and genome-wide methylation. Some studies (4/11) investigated methylation variation in pre-defined sites with quantitative methylation-specific PCR. These studies revealed *TNFSF11, CDH23* and SLC*26A4* genes specifically have significant variation in methylation within gene encoding regions that is associated with audiologically tested variation in hearing loss. Other studies (7/11) performed whole-genome methylation array, reduced representation bisulfite sequencing (RRBS) or whole-genome bisulfite sequencing (WGBS). These studies identified significant variation in DNA methylation within gene promoter regions including *DUSP4, C21orf58, ALG10, C3, LCK, GBX2.* However, female-only studies highlighted a different subset of genes to be significantly differentially methylated, namely, *TCF25, FGFR1, POLE, P2RX2, KCNQ5, ERBB3* and *SOCS3*. One study reported the significant differential methylation was detected in genes that were related to the concurrent disease, Type 2 Diabetes Mellitus, with no hearing loss genes being affected [11].

All animal studies, summarized in Table 3, were conducted in China on adult mice or rats except for one study which focused on rat offspring. The methodological approach for these studies fit into two main categories, site-specific methylation studies and histology paired with immunofluorescence. Site-specific DNA methylation assays (2/4) identified promoter hypermethylation of *gjb2* gene in rats with inner hair cell damage induced by hypoxia. Immunofluorescence studies (2/4) independently showed that, in mice, inhibiting the DNA (cytosine-5)-methyltransferase 1 (*dnmt1*) enzyme can improve noise-induced hearing loss and promote hair cell regeneration.

**Table 3:** Descriptive features of selected studies which investigated DNA methylation in relation to hearing loss in animal models

Animal studies							
Author/Year	Country	Disease	Design	Sample Size	Species/age	Main objective	Results
Zhang, X., et al. 2023 [13]	China	Induced intermittent hypoxia	Methylation-Specific PCR	*n* = 28	Rats/56 days	Determine whether *uhrf1* can induce the methylation of *gjb2* in cochlea damaged by intermittent hypoxia (IH)	*uhrf1* is highly expressed in IH-injured cochlea/hair cells and induces hypermethylation of *gjb2* when combined with *gjb2*
Zheng, Z., et al. 2021 [14]	China	Noise-Induced Hearing Loss	Histology and Immune Fluorescence	no info	Mice/ 12 weeks	Assessing the effects of DNA methylation on noise-induced hearing loss	Inhibition of *dnmt1* ameliorates noise-induced hearing loss and indicates that *dnmt1* may be a promising therapeutic target
Lin, J., et al. 2018 [15]	China	Prenatal Hypoxia	Methylation-Specific PCR	*n* = 120	Rat Offspring/ No info	Examine *gjb2* promoter methylation in rats exposed to chronic prenatal hypoxia	Significant hypermethylation of CpG sites within the *gjb2* promoter region was found in offspring exposed to hypoxia in utero. In addition, subsequent decrease in *gjb2* expression as well as inner and outer hair cell defects in the organ of Corti of these Rats
Deng, X. and Z. Hu 2020 [16]	China	Chemically Induced Hearing Loss	Histology and Immune Fluorescence	no info	Mice/ 4–6 weeks	Identify if generic DNA methylation inhibitor can regenerate hair cells	Hair cell damage induced by kanamycin was shown to be regenerated via *sox2*-positive supporting cells following exposure to DNA methylation inhibitor. Hence global or specific methylation events may prevent re-generation of damaged hair cells
Deng, X., et al. 2019 [17]	China	Chemically Induced Hearing Loss	Histology and Immune Fluorescence	n = 48	Mice/4–6 weeks	Identify if generic DNA methylation inhibitor can regenerate outer hair cells	DNMT inhibitor may promote hair cell regeneration in a chemically deafened mouse model

The selection criteria additionally identified 8 human studies, where mutations in the *DNMT1 gene* were investigated in relation to hearing loss (Table 4). Methodological approaches for these studies were genotyping or exome sequencing. However, 2/8 of these studies included an additional DNA methylation assay in conjunction. In particular, 4/8 studies independently confirmed functional mutations in the *DNMT1* gene to be strongly associated with autosomal dominant cerebellar ataxia, deafness and narcolepsy (ADCA-DN) with 3/8 of these studies consistently showing mutations within exon 21 of *DNMT1* to be found in ADCA-DN patients or children of ADCA-DN patients. One study further highlighted 82 significantly hypermethylated regions in ADCA-DN patients with exon 21 *DNMT1* mutations; however, it was concluded that further work with a more robust dataset would be needed to evaluate the importance of these hypermethylated regions to hearing loss. Patients with hereditary sensory and autonomic neuropathy (HSAN1), noise-induced hearing loss (NIHL), dementia and cognitive decline have all been identified to carry *DNMT1* mutations within various locations. While 6/8 studies had a small sample size of *n* = 6 or less, only two cohort studies have been identified in the search. The largest cohort study (*n* = 1053) conducted in Chinese adults showed polymorphisms in both *DNMT1* and *DNMT3A* that were implicated in noise-induced hearing loss (NIHL).

**Table 4:** Descriptive features of selected studies which investigated *DNMT1* mutations in humans with relation to hearing loss

Human DNMT1 mutation studies									
Author/Year	Country	Disease	Design	Covariates	Sample Size	Sex/Age	Main Objective	Results	Rep
Davis K. N. 2023 [18]	Italy & Sweden	ADCA-DN	Ips and Ins cells derived from patient and control fibroblasts examined via genome-wide target capture sequencing, pyrosequencing and RNA-seq	NA	*n* = 6 case = 3 ctrl = 3	M Only/ 32–57	ips and ins cells derived from ADCA-DN patients were assessed for DNA methylation and gene expression changes	Functional *DNMT1* mutations in ADCA-DN induce global, and cell type-specific, changes to patterns of DNA methylation and gene expression	No
Ding E et al. 2018 [19]	China	NA	SNP selection from Genotyping	Smoking & Alcohol	*n* = 1054 case = 527 ctrl = 527		Explore effects of *DNMT1* and *DNMT3A* polymorphisms on susceptibility to NIHL in Chinese workers	Haplotypes AGGG and TGGA (rs7578578-rs749131-rs1550117-rs2228611) & GG genotype at rs749131 and the AG/GG genotypes at rs1550117 and rs2228611 associated with higher risk of NIHL	No
Klein C.J. 2011 [20]	Europe	HSAN1 with dementia and SNHL	Exome Sequencing	NA	*n* = 63		Show mutations in *DNMT1* cause both central and peripheral neurodegeneration in one form of HSAN1 with dementia and hearing loss	Mutation c.A1484G (p.Tyr495Cys) and triple nucleotide change c.1470TCC-1472ATA (p.Asp490Glu-Pro491Tyr) within targeting sequence of DNMT1 in HSAN1) with dementia and hearing loss patients	No
Menon P.J. 2023 [21]	Ireland	Progressive deafness, mild cognitive decline and apathy	WES	NA	*n* = 1 case = 1	M/ 42	Examination of clinical patient	Novel variant found overlapping HSN1E-cerebellar phenotype	No
Moghadam KK et al. 2014 [22]	Italy	NA	Genetic Tests	ADCA-DN parent	*n* = 2 case = 2	F/ 23–28	Report the clinical picture of two asymptomatic daughters of a patient with ADCA-DN due to *DNMT1* mutation	Mutation on exon 21 of the *DNMT1* gene–p.Ala570Val (RefSeq NM_001130823.1: c.1709G.A)	No
Winkelmann J et al., 2012 [23]	Italy, USA & Sweden	ADCA-DN	WES	NA	*n* = 5 case = 5	No info/ 29–47	To identify the cause of ADCA-DN	Mutations located in exon 21 of *DNMT1* and in very close spatial proximity, suggesting distinct phenotypes depending on mutation location within this gene	No
Zheng W et al. 2018 [24]	China	Sporadic cerebellar ataxia, multiple motor and sensory neuropathy, hearing loss and psychiatric manifestations	WES	NA	*n* = 1	F/ 38	Report a Chinese patient with suspected HSAN1E, confirmed by exome sequencing	Novel heterozygous missense variant, c 1618 T > A (p. Y540N) in exon 20 of the *DNMT1*, which is associated with HSAN1E	No
Kernohan, K. D., et al. 2016 [25]	Canada, Ontario	ADCA-DN	Familial segregation study. 450 K Methylation Array	NA	n = 6		Describe a family with ADCA-DN caused by mutations in *DNMT1* and assess the DNA methylation profile of these individuals	A heterozygous *DNMT1* variant, c.1709C > T [p.Ala570Val] by Sanger sequencing pathogenic for ADCA-DN segregated with disease in the family. Eighty-two significantly hypermethylated regions with further work required to understand significance of these regions to the disease	No

*ADCA-DN* Autosomal dominant cerebellar ataxia, deafness, and narcolepsy, *Ips* Induced Pluripotent Cells, *Ins* Induced Neuronal Cells,*HSAN1* hereditary sensory and autonomic neuropathy, *SNHL* Sensory Neuronal Hearing Loss, *NIHL* noise-induced hearing loss, *WES* whole exome sequencing

### Risk of bias analysis

For human studies, the detailed analysis based on the seven domains of ROBINS-E is summarized in Table S1. According to this, 11 studies had a low risk of bias [11–14], 5 studies had a moderate risk of bias within at least one domain [15–19], and 4 studies were evaluated to have a high risk of bias within at least one domain [20–23].

Animals studies risk of bias analysis is summarized in Table S2.

## Discussion

This review was aimed to summarize emerging evidence which suggests DNA methylation may play an important role in a variety of conditions that are associated with hearing loss. We conducted a systematic review of all available literature where DNA methylation was investigated in conjunction with audiological testing in the context of aging as well as pathologies where hearing loss is a major aspect of the disease. We included a total of 25 studies, 12 performed in patients with concurrent presence of a relevant pathology (Tables 1, 2), 5 conducted in induced hearing loss animal models (Table 2) and 8 which focused on genetic screening of *DNMT1* specifically (Table 3). Overall these studies showcase an association between DNA methylation and hearing loss with a strong need for larger, more robust datasets that may aid in developing a fuller understanding of the molecular mechanisms and key gene pathways that encompass hearing loss.

ARHL is a complex disorder resulting from the interaction of common and rare genetic variation with environmental exposure. Aging is associated with the additive effect of lifestyle and environmental factors both of which can be influenced by DNA methylation. In addition, there is already a large body of evidence which showcases the importance of DNA methylation in aging [24]. This may explain why our search criteria found most methylation studies in humans (6/12) have been performed in individuals with ARHL (Table 1). Despite our selection criteria including 50% of studies being ARHL focused, only one of these studies had partially replicated their findings in an independent cohort (*n* = 203) [22]. The lack of replication is an important consideration for future study designs since the potential clinical relevance of site-specific or global alterations in DNA methylation cannot be correlated to relevant hearing loss contexts without the added evidence of replication cohorts. Hence, at present there is strong association between variation in promoter methylation of genes such as *CDH23, SLC26A4, TCF25* and *POLE* in women with ARHL; however, further investigations in larger cohorts and replication experiments are needed to consolidate these findings. In addition, gender-specific considerations are especially important in DNA methylation studies. Different DNA methylation patterns have been identified across several tissues in men compared to women. These differences have been attributed to mirror gender-specific transcriptomic and proteomic profiles [25–27]. For example, comprehensive description of sex differences in DNA methylation changes with respect to aging in a whole blood dataset consisting of over 400 healthy subjects identified a number of regions where age-related increase in methylation variability was 15 times higher in males compared to females [28]. Hence future studies may benefit greatly by accounting for gender-specific studies. Furthermore, although there is substantial evidence linking aging and DNA methylation, whether there is a relationship between onset of hearing loss and DNA methylation remains largely under explored. In future investigation related to understanding whether DNA methylation across hearing loss genes may contribute to loss of function or missense variation would be beneficial (Table 2).

Secondly, a wide variety of genome-wide DNA methylation assays have been utilized in the studies which were identified in our selection criteria. The main difference in these techniques, namely, RRBS, 27 K, 450 K, 850 K methylation arrays as well as WGBS, is the scope of genes and relevant genomic regions that can be assayed for differential methylation patterns simultaneously. While 450 K methylation array encompasses a wider range of genes compared to 27 K methylation arrays, RRBS includes all genes but only if they have CpG-rich regions as opposed to WGBS which encompasses all cytosine residues across the entire genome. This makes cross-comparisons between studies difficult since analysis strategies vary greatly for data acquired from various upstream assays. For example, *P2RX2, KCNQ5, ERBB3* and *SOCS3* genes were identified as having significant differential methylation in an ARHL female cohort in Tunisia [20] where RRBS was performed. However, significant differential methylation was identified within a different set of genes, *TCF25, FGFR1, POLE*, in a female only cohort of ARHL in the UK where a 27 K methylation array was used. This same study, from the TwinsUK registry, then confirmed differential methylation profiles in promoters of *TCF25* and *POLE* in a second cohort using a 450 K methylation array [22]. While both studies were focused on ARHL, due to inconsistency in study design, it is difficult to formulate the impacts of DNA methylation on ARHL in the context of ethnicity and environment. The genes identified in this study, *TNC25* and *POLE*, have been implicated previously in hearing loss. However, a follow-up study assessing abnormalities in mRNA/protein expression in relevant hearing loss cohorts using the same sample material would have further consolidated these findings. Hence more comprehensive studies are necessary to develop our understanding of the impact of DNA methylation in hearing loss.

Therefore, more cohort studies which encompass a standardized study design will aid immensely in growing our understanding of the potential role of DNA methylation in hearing loss as well as whether these changes are gender specific.

Nevertheless, reports of significant differential methylation in *TCF25* and *POLE* genes are interesting findings due to the localization of the proteins they encode. *TCF25* is a transcription factor, member of the ribosome-associated quality control complex, comprising *TCF25, LTN1* and *NEMF* genes; this complex is able to identify protein products from unproductive translation events, targeting them for degradation [29]. The gene is widely expressed in the mouse cochlear epithelium in both sensory and supporting cells [30]. *POLE* encodes a core catalytic subunit of DNA polymerase epsilon, involved in DNA repair and chromosomal DNA replication. Conversely to TCF25, RNAseq data from mice indicate that POLE is restricted to cochlear hair cells, particularly during development [31] Since differential DNA methylation patterns are known to affect gene expression patterns especially when present at gene promoter regions, further investigation is warranted to see how molecular mechanisms may be impacted in hearing loss through differential methylation.

The role of DNA methylation on rare diseases such as monogenic forms of sensorineural hearing loss (SNHL) has been seldom studied, the only exception being mutations in the *DNMT1* gene, that it is associated with ADCA-DN syndrome. Since functional mutations in *DNMT1* have been identified in patients with ADCA-DN, this provides a strong premise for further assessing global DNA methylation patterns in these patients. Only one of these studies further investigated global DNA methylation patterns [23]. The study concluded further work with a more robust dataset is needed to make conclusive remarks. In addition, 450 K is an older assay with advancements such as 850 K methylation arrays as well as WGBS now more readily available than before. Hence a combination of replication cohorts, larger datasets with more robust methodologies has the potential to greatly improve our understanding of the possible role and related molecular mechanisms of DNA methylation in rare diseases such as monogenic forms of SNHL.

Several research areas remain unexplored in hearing loss methylation studies, such as non-CpG methylation [6]. Non-CpG methylation has recently been attributed to allowing evolution of higher complexity in brain function for vertebrate species [32]. Furthermore, the largest cohort study (*n* = 1053) included in our review which investigated NIHL, found polymorphisms in both DNMT1 and DNMT3A to be significant in their cohort [33]. DNMT3A has an emerging role for instigating non-CpG methylation on the genome during brain development [34]. Since hearing loss conditions are often associated with pathologies which can lead to cognitive decline, it may be a useful strategy to consider whether non-CpG methylation may be involved in certain hearing loss conditions.

From the 12 human studies, although many did not account for underlying genetic variation within their respective cohorts, 2/12 studies investigated whether specific genetic variants may be linked to an altered DNA methylation status. Both studies highlighted an association between the presence of specific polymorphisms with differential DNA methylation and subsequent differential gene expression. A recent study has identified 11.2 million unique SNP–CpG associations in peripheral blood taken from 3799 Europeans and 3,195 South Asian samples. The study presented strong evidence regarding the genetic regulation of DNA methylation [35]. Hence studies which account for genetic variation in patients with confirmed hearing loss would be beneficial in the future.

In future it will also be useful to stratify subjects into high- or low-frequency hearing loss subsets. At present, not all study designs address this as an additional layer of complexity which may impact DNA methylation patterns detected.

This discussion is limited to studies which included audiometric assessment in their study design. Although this is an important criterion for assessing relationships between DNA methylation and hearing loss, we cannot ignore that studies which may have addressed this question from a different perspective may also contribute insightful findings which were beyond the scope of this review.

## Conclusions

Overall, the literature collectively provides some evidence, suggesting variation in DNA methylation may play an important role in hearing loss, particularly in ARHL. Hearing ability is associated with methylation profile in the promoter of *TCF25* and *POLE* genes in ARHI.

Epigenetic research should produce larger, more robust datasets where global DNA methylation patterns are investigated thoroughly within the context of standardized study designs. Furthermore, gender-specific cross-study comparisons are needed for insightful knowledge on the role of DNA methylation in hearing loss processes.

## Supplementary Information

Additional file1 (DOCX 51 KB)

## Data Availability

No datasets were generated or analyzed during the current study.
