# Correction: A systematic review on the contribution of DNA methylation to hearing loss

**DOI:** 10.1186/s13148-024-01733-8

**Published:** 2024-09-16

**Authors:** Vibha Patil, Patricia Perez-Carpena, Jose A. Lopez-Escamez

**Affiliations:** 1 Meniere’s Disease Neuroscience Research Program, Faculty of Medicine and Health, School of Medical Sciences, The Kolling Institute, University of Sydney, Rm 611024, Level 11 Kolling Institute | 10 Westbourne St, St Leonards, Sydney, NSW 2064 Australia; 2 Division of Otolaryngology, Department of Surgery, Instituto de Investigación Biosanitaria, Ibs.Granada, Universidad de Granada, Granada, Spain; 3 Otology & Neurotology Group CTS495, Instituto de Investigación Biosanitaria, Ibs.GRANADA, Universidad de Granada, Granada, Spain; 4 Sensorineural Pathology Program, Centro de Investigación Biomédica en Red en Enfermedades Raras, CIBERER, Madrid, Spain; 5 Department of Otolaryngology, Hospital Universitario San Cecilio, Instituto de Investigacion Biosanitaria, Ibs.GRANADA, Granada, Spain

**Correction: Clinical Epigenetics (2024) 16:88** 10.1186/s13148-024-01697-9

Following publication of the original article [1], the authors noticed the error in Fig. 1. The typesetter has inadvertently processed the flowchart with a missing box in the figure. The correct Fig. 1 has been presented with this erratum.

**Fig. 1:**
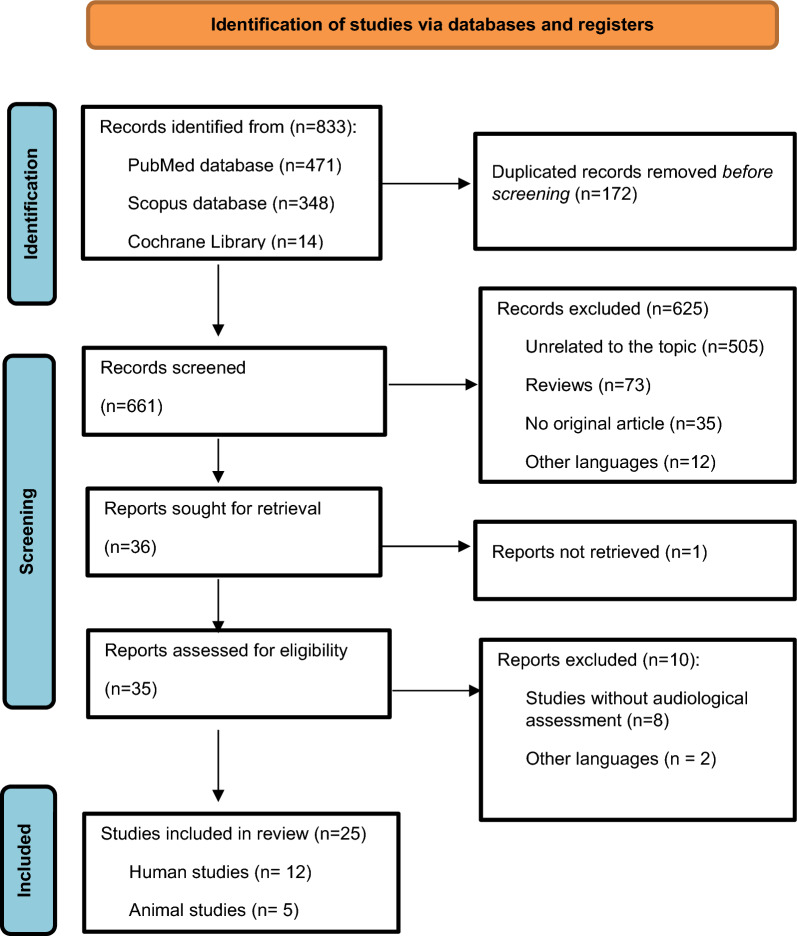
Flow diagram for the DNA methylation study selection

The original article has been corrected.

## References

[1] Patil V, Perez-Carpena P, Lopez-Escamez JA. A systematic review on the contribution of DNA methylation to hearing loss. Clin Epigenet. 2024;16:88. 10.1186/s13148-024-01697-9 PMC11227199 38970134

