# Supplementary material for: A systematic review on the contribution of DNA methylation to hearing loss
Source: Clin Epigenetics. 2024 Jul 5;16:88. doi: 10.1186/s13148-024-01697-9 (PMC11227199; doi:10.1186/s13148-024-01697-9)
Supplement: Supplementary file 1 — Additional file1 (DOCX 51 KB) [file 13148_2024_1697_MOESM1_ESM.docx]

**Supplementary Tables**

Table S1: Risk of bias assessment in human studies, according to the Risk of Bias in Non-randomized Studies of Exposures (ROBINS-E) tool. Seven domains of bias were assessed, and each one was assigned a colour code (green: low; yellow: moderate; red: high; black: very high risk of bias; white: domain not applicable to this study).

|  | **Reference** | **Study Design** | **D1** | **D2** | **D3** | **D4** | **D5** | **D6** | **D7** |
| --- | --- | --- | --- | --- | --- | --- | --- | --- | --- |
| **Human studies** | Bouzid, A., et al. 2022 (21) | Case Control |  |  |  |  |  |  |  |
|  | Bouzid, A., et al. 2018 (22) | Case Control |  |  |  |  |  |  |  |
|  | Bouzid, A., et al. 2018 (13) | Case Control |  |  |  |  |  |  |  |
|  | Brown, A. L., et al. 2017 (23) | Cross Sectional Population Based Study |  |  |  |  |  |  |  |
|  | Flook, M., et al. 2021 (24) | Cross Sectional Population Based Study |  |  |  |  |  |  |  |
|  | Guo, L., et al. 2023 (25) | Cross Sectional Population Based Study |  |  |  |  |  |  |  |
|  | Hao, J., et al. 2018 (10) | Cross Sectional Population Based Study |  |  |  |  |  |  |  |
|  | Kuo, P. L., et al. 2021 (26) | Cross Sectional Population Based Study |  |  |  |  |  |  |  |
|  | Lassaletta, L., et al. 2006 (27) | Cross Sectional Population Based Study |  |  |  |  |  |  |  |
|  | Wolber, L. E., et al. 2014 (12) | Cross Sectional Population Based Study |  |  |  |  |  |  |  |
|  | Xu, J., et al. 2017 (28) | Case Control |  |  |  |  |  |  |  |
|  | Xu, L., et al. 2020 (29) | Case Control |  |  |  |  |  |  |  |
| ***DNMT1* mutations studies** | Davis 2023 (35) | Studies in Families |  |  |  |  |  |  |  |
|  | Ding et al. 2018 (19) | Case Control |  |  |  |  |  |  |  |
|  | Klein 2011 (36) | Cross Sectional Population Based Study |  |  |  |  |  |  |  |
|  | Menon 2023 (37) | Case Report |  |  |  |  |  |  |  |
|  | Moghadam et al. 2014 (38) | Studies in Families |  |  |  |  |  |  |  |
|  | Winkelmann et al, 2012 (39) | Studies in Families |  |  |  |  |  |  |  |
|  | Zheng et al. 2018 (40) | Case Report |  |  |  |  |  |  |  |
|  | Kernohan et al. 2016 (17) | Studies in Families |  |  |  |  |  |  |  |

Table S2: SYRCLE’s risk of bias tool for animal studies. The risk of bias was assessed across 10 Items of potential bias within each animal study included in this systematic review. All studies had a low risk of bias in Items 1,2,6,8,9 and 10. Item 3 had a high risk of bias for all studies since the intervention or induced exposure was not blinded. The risk of bias in items 4,5 and 7 are unclear due to a lack of information provided in the methodology.

| **Animal Study** | **Item 1** | **Item 2** | **Item 3** | **Item 4** | **Item 5** | **Item 6** | **Item 7** | **Item 8** | **Item 9** | **Item 10** |
| --- | --- | --- | --- | --- | --- | --- | --- | --- | --- | --- |
| Zhang, X., et al. 2023 (30) | Low | Low | High | Unclear | Unclear | Low | Unclear | Low | Low | Low |
| Zheng, Z., et al. 2021 (31) | Low | Low | High | Unclear | Unclear | Low | Unclear | Low | Low | Low |
| Lin, J., et al. 2018 (32) | Low | Low | High | Unclear | Unclear | Low | Unclear | Low | Low | Low |
| Deng, X. and Z. Hu 2020 (33) | Low | Low | High | Unclear | Unclear | Low | Unclear | Low | Low | Low |
| Deng, X., et al. 2019 (34) | Low | Low | High | Unclear | Unclear | Low | Unclear | Low | Low | Low |
